# Low genetic variation of invasive *Fallopia* spp. in their northernmost European distribution range

**DOI:** 10.1002/ece3.3703

**Published:** 2017-12-05

**Authors:** Anne‐Kari Holm, Abdelhameed Elameen, Benedikte W. Oliver, Lars O. Brandsæter, Inger S. Fløistad, May B. Brurberg

**Affiliations:** ^1^ Norwegian Institute of Bioeconomy Research Ås Norway; ^2^ Norwegian University of Life Sciences Ås Norway

**Keywords:** amplified fragment length polymorphism, clonal reproduction, giant knotweed, hybrid knotweed, invasive alien species, Japanese knotweed, simple sequence repeats

## Abstract

Knowledge about the reproduction strategies of invasive species is fundamental for effective control. The invasive *Fallopia* taxa (Japanese knotweed *s.l*.) reproduce mainly clonally in Europe, and preventing spread of vegetative fragments is the most important control measure. However, high levels of genetic variation within the hybrid *F*. × *bohemica* indicate that hybridization and seed dispersal could be important. In Norway in northern Europe, it is assumed that these taxa do not reproduce sexually due to low temperatures in the autumn when the plants are flowering. The main objective of this study was to examine the genetic variation of invasive *Fallopia* taxa in selected areas in Norway in order to evaluate whether the taxa may reproduce by seeds in their most northerly distribution range in Europe. *Fallopia* stands from different localities in Norway were analyzed with respect to prevalence of taxa, and genetic variation within and between taxa was studied using amplified fragment length polymorphism (AFLP). Taxonomic identification based on morphology corresponded with identification based on simple sequence repeats (SSR) and DNA ploidy levels (8× *F*. *japonica*, 6× *F*. × *bohemica* and 4× *F. sachalinensis*). No genetic variation within *F*. *japonica* was detected. All *F*. × *bohemica* samples belonged to a single AFLP genotype, but one sample had a different SSR genotype. Two SSR genotypes of *F. sachalinensis* were also detected. Extremely low genetic variation within the invasive *Fallopia* taxa indicates that these taxa do not reproduce sexually in the region, suggesting that control efforts can be focused on preventing clonal spread. Climate warming may increase sexual reproduction of invasive *Fallopia* taxa in northern regions. The hermaphrodite *F*. × *bohemica* is a potential pollen source for the male‐sterile parental species. Targeted eradication of the hybrid can therefore reduce the risk of increased sexual reproduction under future warmer climate.

## 1 INTRODUCTION

**Figure 1:**
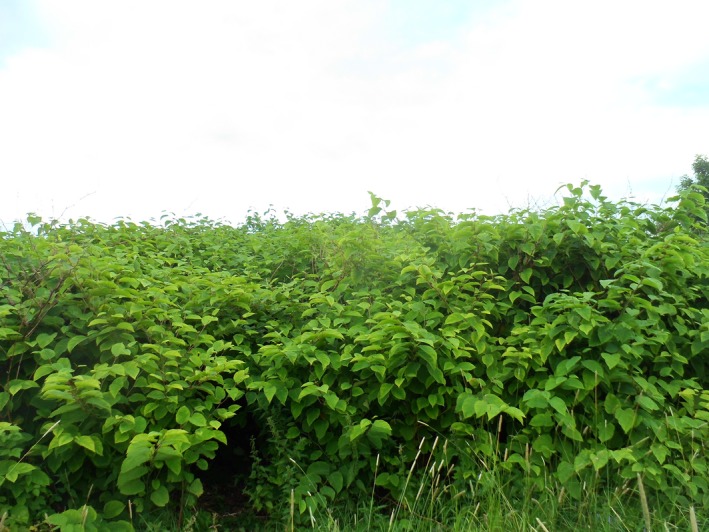
The invasive *Fallopia* taxa form large monocultures that displace native species, reduce biodiversity, and degrade habitats. The picture shows a stand of *Fallopia* × *bohemica*, photographed in July in Ås, Norway

The invasive *Fallopia* taxa, belonging to the Polygonaceae family, are among the most problematic invasive alien plants in Europe and North America (recently summarized by Robinson, Inger, Crowley, and Gaston (2017)). These taxa develop extensive rhizome systems and form large monocultures that displace native species, reduce biodiversity, and degrade habitats (Aguilera, Alpert, Dukes, & Harrington, 2010) (Figure 1). Spread of rhizome and stem fragments by humans and water streams are the main means of spread in Europe (Bailey, Bímová, & Mandák, 2009). However, hybridization between the taxa is common, and reproduction by seeds may be more important than previously assumed (Bzdęga et al., 2016; Hollingsworth & Bailey, 2000b; Krebs et al., 2010; Mandák, Bímová, Pyšek, Štěpánek, & Plačková, 2005; Tiébré, Bizoux, Hardy, Bailey, & Mahy, 2007). Sexual reproduction increases genetic diversity and produces new genotypes that may be better adapted to the environment and more tolerant to chemical or biological control treatments. Furthermore, seed dispersal can also increase spread and consequently complicate management.

Three *Fallopia* taxa are invasive in Europe: *Fallopia japonica* (Houtt.) Ronse de Craene var. *japonica* (here referred to as *F*. *japonica*), *F. sachalinensis* (F. Schmidt ex Maxim) Ronse de Craene, and their hybrid, *F*. × *bohemica* (Chrtek and Chrtková) J. P. Bailey. All three taxa are rhizomatous perennials that are polyploid, gynodioecious, and generally self‐incompatible. *Fallopia japonica* and *F. sachalinensis* are native to East Asia and were introduced to Europe as ornamental and fodder plants in the mid‐1800s (Bailey, 1994). The two species escaped cultivation and have hybridized in the introduced range to produce *F*. × *bohemica* (Bailey, 1994). In Norway, Schübeler reported in 1883 that *F*. *japonica* had been cultivated as an ornamental plant for several years and had been distributed north to Bodø (67°N) (Schübeler, 1883). The earliest herbarium records of *F. sachalinensis* and *F*. × *bohemica* in Norway are dated 1935 and 1964, respectively (Fremstad & Elven, 1997). *Fallopia* × *bohemica* is sometimes misidentified as either of the parental species, and its distribution is likely underestimated (Bailey & Wisskirchen, 2004). The hybrid can be more invasive (Parepa, Fischer, Krebs, & Bossdorf, 2014) and difficult to control (Bímová, Mandák, & Pyšek, 2001) than the parental species.

The genetic variation of the invasive *Fallopia* taxa has previously been studied in the UK (Hollingsworth & Bailey, 2000b; Pashley, Bailey, & Ferris, 2007), parts of mainland Europe (Bzdęga et al., 2016; Krebs et al., 2010; Mandák et al., 2005; Tiébré, Bizoux, et al., 2007), and in North America (Gammon & Kesseli, 2010; Gaskin et al., 2014; Grimsby, Tsirelson, Gammon, & Kesseli, 2007; Groeneveld, Belzile, & Lavoie, 2014). *Fallopia japonica* is mostly present as a single, male‐sterile, octoploid genotype in its introduced range. However, a low level of genetic variation was detected within *F*. *japonica* in Poland (Bzdęga et al., 2012, 2016). By use of amplified fragment length polymorphism (AFLP), Bzdęga et al. (2016) detected up to five different genotypes, and a mean level of polymorphism up to 0.45%, within populations of *F*. *japonica*. Furthermore, Gammon and Kesseli (2010) detected eight haplotypes of *F*. *japonica* in North America by use of a chloroplast single nucleotide polymorphism. The variation found within *F*. *japonica* may be due to multiple introductions and postintroduction mutations (Bzdęga et al., 2016; Gammon & Kesseli, 2010). *Fallopia sachalinensis* and *F*. × *bohemica* are both present as male‐sterile and hermaphrodite individuals in Europe. *Fallopia* × *bohemica* can be partially to fully fertile. The two taxa can reproduce sexually within taxa, as well as by hybridization and backcrossing, and genetic variation is found within both taxa. They have various ploidy levels, of which tetraploid *F. sachalinensis* and hexaploid *F*. × *bohemica* are the most common (Mandák et al., 2003; Tiébré, Bizoux, et al., 2007). Particularly high levels of genetic and cytological variation within *F*. × *bohemica* indicate that hybridization and sexual reproduction play important roles in the development and spread of this species (Bzdęga et al., 2016; Gaskin et al., 2014; Mandák et al., 2005).

Due to the absence of hermaphrodite *F*. *japonica* plants in Europe, this species produces seeds only by hybridization with related taxa, mainly; *F. sachalinensis*,* F*. × *bohemica*, and *F. baldschuanica*. Most seeds of *F*. *japonica* examined in Europe have been fathered by *F*. × *bohemica* or *F. baldschuanica* (Bailey, 1994; Tiébré, Vanderhoeven, Saad, & Mahy, 2007). The latter is a diploid, commonly grown ornamental, but its hybrid with *F*. *japonica*,* F*. × *conollyana* (2*n* = 54), very rarely establishes in nature (Bailey & Spencer, 2003). Another species involved in hybridization with the invasive *Fallopia* taxa is *F. japonica* var. *compacta*. This tetraploid, dwarf variety is generally rare in Europe (Bailey, 1994; Mandák et al., 2005; Tiébré, Bizoux, et al., 2007), but is important in hybridization with *F. sachalinensis* in certain regions (Bailey et al., 2009).

Although the seeds of invasive *Fallopia* taxa germinate and grow into viable plants when grown under optimal conditions in experiments, observations of seedlings are not very common in nature in Europe (Funkenberg, Roderus, & Buhk, 2012; Mandák et al., 2005; Tiébré, Vanderhoeven, et al., 2007). This indicates that seedling establishment in the field requires certain environmental conditions. Seedling establishment is negatively affected by dry conditions, late spring frost, and competition for light (Forman & Kesseli, 2003; Funkenberg et al., 2012). In addition, seeds are susceptible to fungal attacks and predation by birds (Bailey & Spencer, 2003; Engler, Abt, & Buhk, 2011).

Information about the reproduction modes of the invasive *Fallopia* taxa in more northern regions of Europe is lacking. In Norway in northern Europe, it is assumed that late flowering (end of August to October) combined with a cold autumn climate and early frosts limits the production of mature seeds (Fremstad & Elven, 1997). However, seed production has been observed in these taxa in Norway (personal observation), and one record of *F*. × *conollyana* from Stavanger in southwest Norway (Fremstad & Elven, 1997) indicates that occasional establishment from seed may occur.

The objective of this study was to examine the genetic diversity of *F*. *japonica*,* F. sachalinensis*, and *F*. × *bohemica* in selected areas of Norway, in order to evaluate whether the taxa reproduce sexually in their northern distribution range in Europe. This is the first genetic diversity study of these taxa in Norway and the hitherto northernmost study on this subject.

## 2 MATERIALS AND METHODS

### 2.1 Sample collection

**Figure 2:**
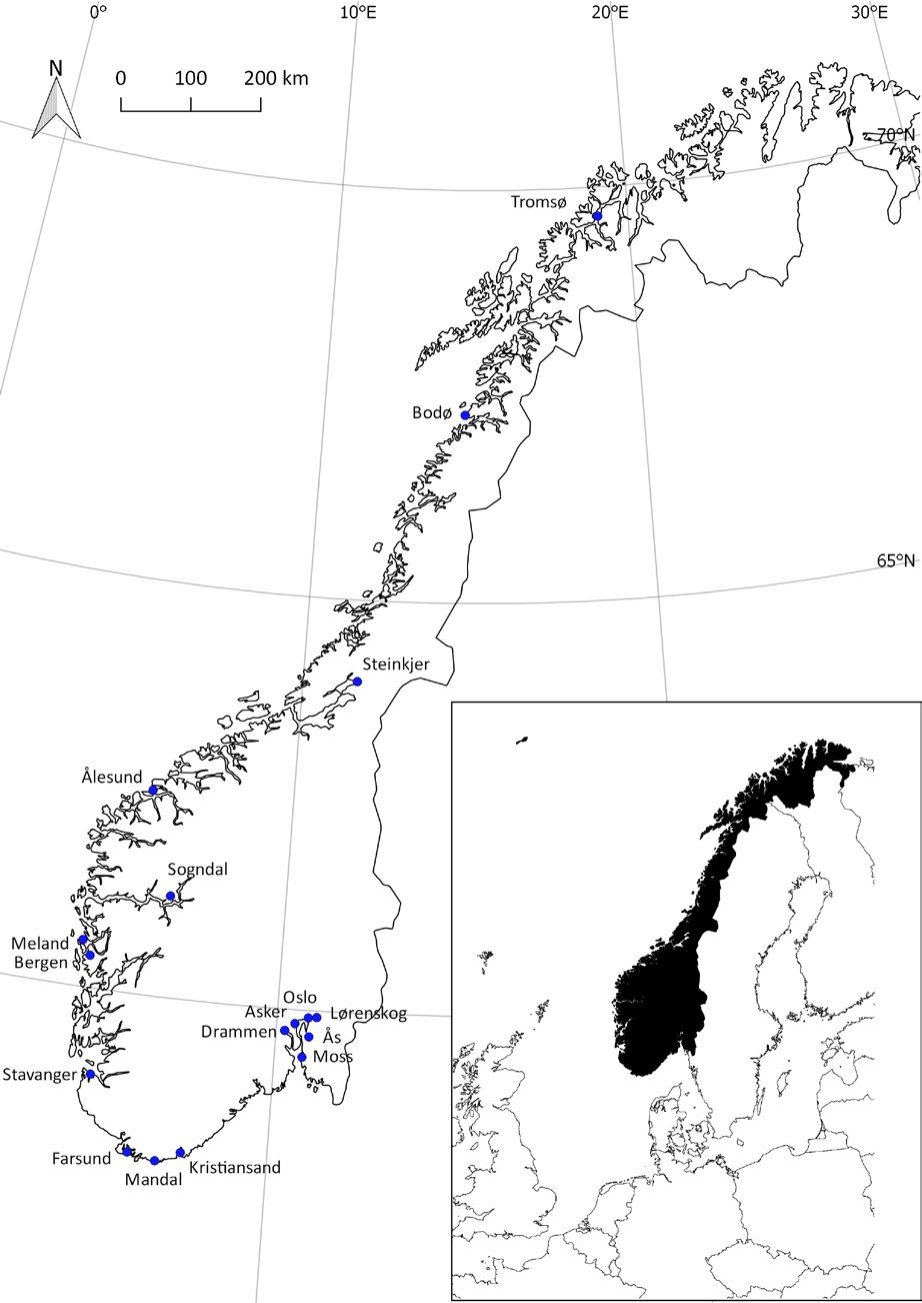
Map showing the geographical positions of the localities where samples of invasive *Fallopia* taxa were collected in Norway

Sampling localities were situated in lowland coast and fjord regions of Norway. The southernmost locality had a latitude of 58.0°N, and the northernmost locality had a latitude of 69.6°N (Figure 2). According to the Köppen‐Geiger climate classification system (Peel, Finlayson, & McMahon, 2007), the Norwegian sampling area has a temperate to cold climate, with no dry seasons, and with warm to cold summers.

One sample per stand was collected from 145 stands of invasive *Fallopia* taxa in 17 different localities in Norway during July–October in 2012–2014. Each sample was collected from randomly chosen, putatively monospecific, isolated stands separated by a minimum of 50 meters. A stand is here defined as a discrete grouping of *Fallopia* shoots with at least seven meters distance from other such groupings. Seven meters is the length the rhizome system can extend from a parent plant (Child & Wade, 2000).

The number of samples collected from each locality varied (Table 1), which reflects variation in sampling intensity and not necessarily the abundance of invasive *Fallopia* plants.

**Table 1:** Number of samples of invasive *Fallopia* taxa collected from different localities in Norway

Locality	FJ	FB	FS	Total
Moss	21	5		26
Ås	17	2		19
Lørenskog	1	1		2
Oslo	8	12		20
Asker		1		1
Drammen	10	5	2	17
Kristiansand	5			5
Mandal	2			2
Farsund	2			2
Stavanger	3			3
Bergen	33			33
Meland	2			2
Sogndal	1			1
Ålesund	3			3
Steinkjer		3		3
Bodø	1			1
Tromsø	1	3	1	5
Total	110	32	3	145

FJ, *F*. *japonica*; FB, *F*. × *bohemica*; FS, *F. sachalinensis*.

For morphological identification, some of the largest leaves from the mid to lower part of the main stems were used. For molecular and flow cytometric analysis, young leaves without signs of fungal infections or herbivory were collected from a single shoot in each stand. Leaves were transported fresh in plastic bags to the laboratory. Each sample was divided into two parts; one part was flash frozen using liquid nitrogen and stored at −20°C, the other part was dried in silica gel and stored at room temperature. For 35 samples collected in west Norway, the complete samples were silica‐dried directly after collection due to longer transportation time from the field to the laboratory.

A silica‐dried sample of octoploid *F*. *japonica* from the UK was provided by John Bailey, University of Leicester. This sample was used for comparison with Norwegian samples in all analyses.

### 2.2 Morphological identification

Each sample was assigned to a species based on the diagnostic characters described by Bailey and Wisskirchen (2004). If samples contained flowers, the flowers were examined under a stereomicroscope (Leica MZ125), and sex expression was determined according to Bailey (1994).

### 2.3 Flow cytometric analysis

Flow cytometry allows for rapid determination of the relative DNA content by measuring the intensity of fluorescent light emitted from isolated nuclei that have been stained with a DNA‐specific fluorochrome (Galbraith et al., 1983). Silica‐dried leaves from 124 samples, including the octoploid control sample from the UK, were subjected to flow cytometric analysis for determination of DNA ploidy level. The remaining 22 samples were not analyzed due to lack of silica‐dried leaves. The analysis was performed by Plant Cytometry Services, the Netherlands (http://plantcytometry.nl). The samples were chopped together with the internal standard, *Pachysandra terminalis*, and collected in a DNA buffer with DAPI as the fluorochrome. After sieving, the nuclei were analyzed with CyFlow Space (Partec GmbH, Germany) with a UV High power LED (365).

### 2.4 DNA extraction

DNA was extracted from frozen or silica‐dried leaves using Qiagen's Plant DNeasy Mini Kit (Qiagen Inc., Hilden, Germany) according to the manufacturer's protocol.

### 2.5 Simple sequence repeats analysis

To aid taxonomic identification, all 145 samples were analyzed with the simple sequence repeats (SSR) markers KW2 and KW6, developed by Grimsby et al. (2007). KW6 amplifies a fragment in *F. sachalinensis* and *F*. × *bohemica*, but not in *F*. *japonica* (Grimsby et al., 2007). KW2 amplifies a fragment in all three taxa (Grimsby et al., 2007) and was included in the analysis as a control to verify that the DNA quality was appropriate for PCR amplification and that the samples were from *Fallopia*. PCR mixture and cycling conditions were essentially as described by Grimsby et al. (2007). Amplification result was visualized by running 10 μl PCR product on agarose (1.5%) gel electrophoresis at 90–110 V for approximately 2–3 hr. Approximate fragment size was determined by comparing with a 100 bp DNA ladder (New England Biolabs).

### 2.6 Amplified fragment length polymorphism analysis

The genetic variation within and between the *Fallopia* taxa was examined by use of AFLP analysis (Vos et al., 1995). This robust, reproducible method allows for simultaneous analysis of many polymorphic loci with relatively few primers (Costa, Pereira, Garrido, Tavares‐de‐Sousa, & Espinosa, 2016; Jones et al., 1997). AFLP analysis has previously been used successfully to detect genetic diversity within the invasive *Fallopia* taxa (Bzdęga et al., 2012, 2016; Gaskin et al., 2014). AFLP analysis was performed as previously described (Elameen et al., 2008), but with modifications that included the use of fluorescently labeled primers instead of radioactive labeling. Briefly, 300 ng of genomic DNA was double‐digested with *Eco*RI and the *Mse*I isoschizomer *Tru*1I. Following ligation of the restriction fragments to the adaptors, preamplification PCR was carried out with nonselective primers in a total volume of 25 μl containing 5 μl of fivefold diluted ligation product. Five primer pairs were selected and used to generate AFLP fragments of all of the taxa: *Eco*RI‐GA + *Mse*I‐CA; *Eco*RI‐GA + *Mse*I‐CC; *Eco*RI‐AC + *Mse*I‐CG; *Eco*RI‐AC + *Mse*I‐CC; *Eco*RI‐GA + *Mse*I‐CG. The fluorescently labeled PCR products were analyzed using an ABI3730 DNA Analyzer. One microliter of PCR product was added to a loading buffer containing 8.75 μl Hi‐Di formamide (Applied Biosystems) and 0.25 μl of GeneScan 500 LIZ size standard (Applied Biosystems). The data were collected using the software Data Collection v 2.0 (Applied Biosystems). GeneMapper v 4.1 (Applied Biosystems) was used to derive the fragment lengths of the labeled DNA‐fragments using the known fragment lengths of the LIZ‐labeled marker peaks and scored for the presence (1) or absence (0) of peaks. All peaks were checked manually, and only clear and unambiguous peaks with fluorescence greater than or equal to 100 arbitrary units were entered into a binary data matrix for further analysis.

The AFLP analysis, including DNA extraction, was repeated twice, using all primer combinations, for 36 randomly selected samples (26 *F*. *japonica*, eight *F*. × *bohemica*, and two *F. sachalinensis*). The replicated profiles were compared, and markers with more than 5% errors were removed from the data set.

### 2.7 Genetic analysis

The genetic similarity (GS) was estimated using the Dice coefficient, calculated as GS_*xy*_ = 2*a*/(2*a *+* b *+* *c), where *a* is the number of peaks present in both plant *x* and plant *y*,* b* is the number of peaks present only in plant *x*, and *c* is the number of peaks present only in plant *y* (Dice, 1945). The genetic similarity among the individual plants, based on the presence or absence of amplified fragments, was also calculated by Jaccard's coefficient (Jaccard, 1908).

The similarity matrices obtained were analyzed using the unweighted pair group method with arithmetic mean (UPGMA), as suggested by Sneath and Sokal (1973). The similarity matrices were also used to perform principal coordinate analyses (PCO) to classify and detect potential structure within and among the taxa. The analyses and the dendrogram constructions were performed using NTSYS‐pc software (Rohlf, 2000).

The genetic structure of the *Fallopia* samples was also investigated using the model‐based Bayesian clustering approach of genetic mixture analysis (structure 2.3.4 software) (Falush, Stephens, & Pritchard, 2003). These analyses assume that the clusters (*K*) are at Hardy–Weinberg equilibrium and in linkage equilibrium, but the approach has proven to be robust also for deviations from these assumptions (Falush et al., 2003). Simulations were performed with a data set from *K *=* *1 to *K *=* *6. Six independent runs were conducted to assess the consistency of the results across runs, and all runs were based on 500,000 iterations after a burn‐in period of 100,000 iterations. The method developed by Evanno, Regnaut, and Goudet (2005) was used to identify the number of genetically homogeneous clusters (*K*). For the parameter set in our study, we selected admixture as ancestry model, and the allele frequencies were assumed to be correlated, as it is more reasonable to assume common ancestry of such closely related species. We used a burn‐in period length of 100,000 runs and 500,000 MCMC runs to compute the probability of the data for estimating *K*. Among the six independent runs, the one with the highest Ln Pr (*X*|*K*) value (log probability) was chosen and represented as bar plots. Bar plots of likelihoods and Δ*K*s were made with STRUCTURE HARVESTER (Earl & vonHoldt, 2012).

## 3 RESULTS

### 3.1 Morphological identification

Based on morphological characters, we identified 110 samples as *F*. *japonica*, 32 samples as *F*. × *bohemica*, and three samples as *F. sachalinensis*. The taxonomic composition of collections from the different sampling areas is given in Table 1. Only male‐sterile flowers were observed on *F*. *japonica*. Two *F. sachalinensis* had male‐sterile flowers, while the third sample did not contain flowers. Only hermaphrodite flowers were observed on *F*. × *bohemica*, but one of these had flowers that fit the description of female‐sterile flowers (Bailey, 1994). These flowers had long filaments with well‐filled anthers and poor stigma development. In addition, the inflorescence panicles of the same *F*. × *bohemica* were less compact and with longer branches than the panicles of other *F*. × *bohemica*. Furthermore, the leaves of this plant were more narrowly oblong.

### 3.2 DNA ploidy levels

The ratios between the fluorescence peaks of samples and the internal standard were used to deduce different ploidy levels. Peak ratios of 1.19–1.20, 1.77–2.01, and 2.44–2.83 corresponded to tetraploid, hexaploid, and octoploid ploidy levels, respectively. Samples that based on morphology and SSR analysis had been identified as *F. sachalinensis* were tetraploid, samples identified as *F*. × *bohemica* were hexaploid, and samples identified as *F*. *japonica* were octoploid.

### 3.3 SSR analysis

**Figure 3:**
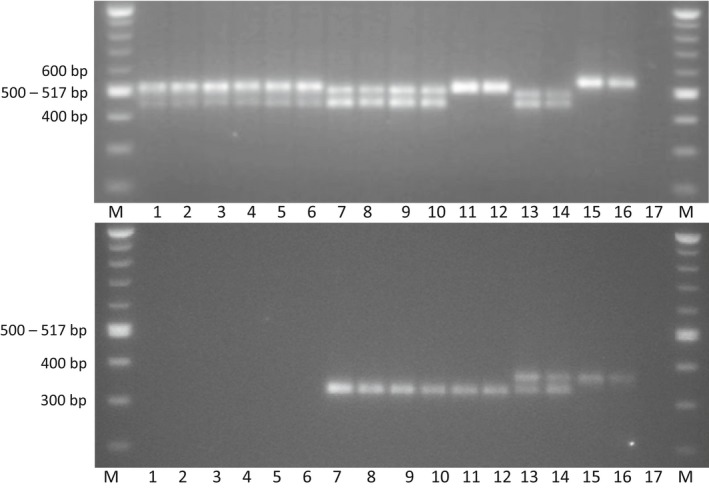
Example of agarose gel with PCR products amplified using primers for the simple sequence repeats (SSR) marker KW2 (upper panel) and KW6 (lower panel). Lane M = 100 bp DNA ladder (New England Biolabs); lane 1 = *Fallopia japonica* sample from the UK; lane 2–6 = *F. japonica* samples from Norway; lane 7–10 = *F*. × *bohemica* samples with the most common SSR genotype; lane 11–12 = two replicates of one *F*. × *bohemica* sample with irregular SSR genotype; lane 13–14 = *F. sachalinensis* samples from Drammen; lane 15–16 = two replicates of one *F. sachalinensis* sample from Tromsø

PCR with the primers for the SSR marker KW2 resulted in amplification of fragments from all the plant samples, indicating that the DNA was of sufficient quality for analysis and that the samples were from *Fallopia*. The KW2 marker primers resulted in two fragments in all samples that, based on morphology, were identified as *F*. *japonica* (fragment size approximately 520 and 460 bp). The *F*. *japonica* samples collected in Norway and the *F*. *japonica* sample from the UK had seemingly identical fragment sizes (Figure 3). For *F. sachalinensis*, two fragments were amplified from the two samples collected in Drammen, southeast Norway (fragment size approximately 500 and 460 bp), while only a single fragment was amplified from the *F. sachalinensis* sample collected in Tromsø, northern Norway (fragment size approximately 550 bp) (Figure 3). Two fragments were amplified from 31 of 32 samples identified as *F*. × *bohemica* (fragment size approximately 510 and 460 bp). A single fragment was amplified from one *F*. × *bohemica* sample collected in Oslo, southeast Norway (fragment size approximately 520 bp) (Figure 3). This was the same sample that also differed from the other *F*. × *bohemica* samples in flower and leaf morphology.

KW6 marker primers resulted in no fragment amplification in the samples that, based on morphology, were identified as *F*. *japonica* (Figure 3). The same primers resulted in amplification of two fragments of approximately 375 and 350 bp in two samples identified as *F. sachalinensis* collected in Drammen, southeast Norway, and a single fragment of 375 bp in one sample identified as *F. sachalinensis* collected in Tromsø, northern Norway (Figure 3). A single fragment of 350 bp was amplified in the 32 samples identified as *F*. × *bohemica* (Figure 3).

### 3.4 Genetic diversity

Amplified fragment length polymorphism analyses of the three taxa (*F*. *japonica*,* F*. × *bohemica* and *F. sachalinensis*), using the five primer combinations, gave a total number of 184 clear polymorphic fragments. These primer combinations were able to distinguish all of the taxa unambiguously, and significant differentiation between the three taxa was found. The results obtained by the Dice coefficient showed that the genetic similarity was as follows: 0.65 between *F*. *japonica* and *F*. × *bohemica*, 0.41 between *F*. × *bohemica* and *F. sachalinensis*, and 0.29 between *F*. *japonica* and *F. sachalinensis*.

**Figure 4:**
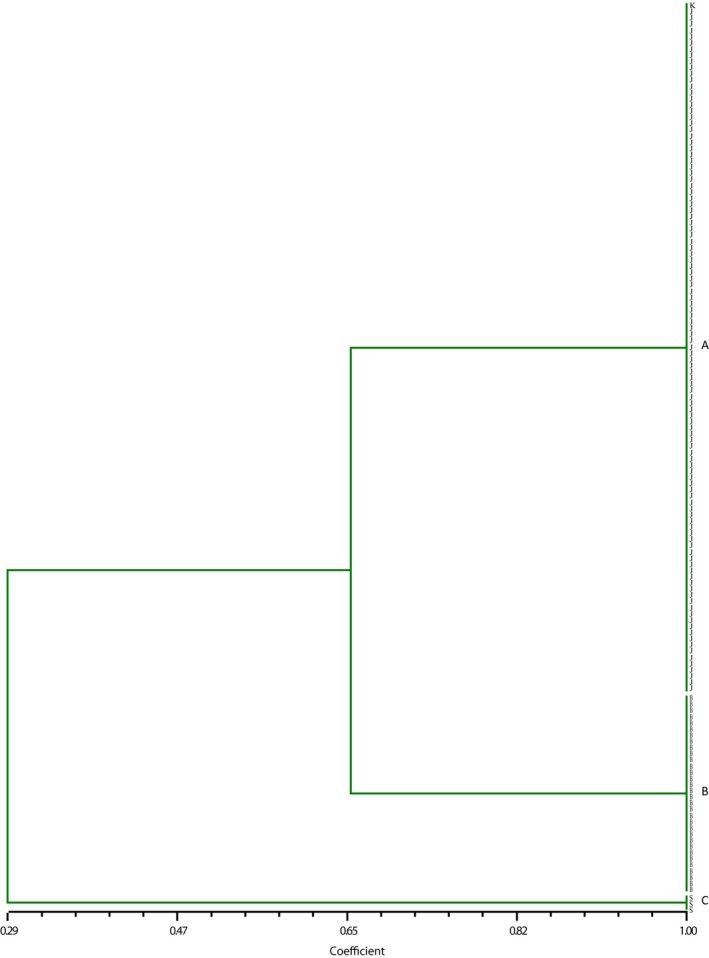
Dendrogram of *Fallopia* samples based on Dice similarity index from 184 polymorphic amplified fragment length polymorphism fragments and unweighted pair group method with arithmetic mean clustering algorithm. Cluster A comprises 111 *Fallopia japonica* (including UK control sample), cluster B comprises 32 *F*. × *bohemica*, and cluster C comprises 3 *F. sachalinensis*

The UPGMA analysis based on the Dice coefficient clustered the three taxa into three separate groups: Cluster A comprised 111 *F*. *japonica*, including the sample from the UK, cluster B comprised 32 *F*. × *bohemica*, and cluster C comprised three**** *F. sachalinensis* (Figure 4). The same clusters were obtained when the AFLP data were analyzed using only three primer combinations (data not shown). Both Dice and Jaccard analyses resulted in the same clusters, hence only the results obtained by the Dice coefficient are presented. The results of the PCO analysis supported the results of the UPGMA analysis (data not shown).

**Figure 5:**
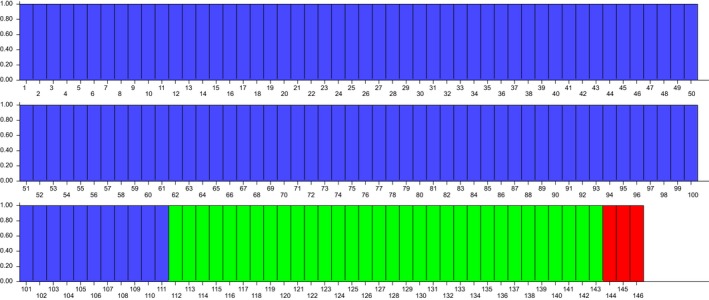
Genetic STRUCTURE bar plot based on amplified fragment length polymorphism analysis of 146 individuals of invasive *Fallopia* taxa. The three clusters correspond to different taxa (111 *F*. *japonica* including control from the UK—blue; 32 *F*. × *bohemica*—green; three *F. sachalinensis*—red)

Structure analysis showed the maximum likelihood distribution *L* (*K*) at the real number of three groups (*K *=* *3). This value was obtained using the value of ad hoc quantity (∆*K*) rather than maximum likelihood value *L* (*K*) as described by Evanno et al. (2005). Structure analysis clustered the *Fallopia* taxa into three main clusters (Figure 5) as for the UPGMA and PCO groupings (data not shown).

The analysis of pairwise distances showed that the greatest genetic distance was as expected between *F*. *japonica* and *F. sachalinensis* (0.71), while the shortest genetic distance was between *F*. *japonica* and *F*. × *bohemica* (0.36) (Table 2).

**Table 2:** Average genetic distances among three invasive *Fallopia* taxa calculated by the pairwise genetic distance method (Excoffier & Smouse, 1994)

Species	*F*. *japonica*	*F*. × *bohemica*	*F. sachalinensis*
*F*. *japonica*	0.00		
*F*. × *bohemica*	0.36	0.00	
*F. sachalinensis*	0.71	0.52	0.00

## 4 DISCUSSION

*Fallopia japonica* was the most frequent of the three *Fallopia* taxa sampled in Norway, followed by *F*. × *bohemica* and *F. sachalinensis*. *F*. *japonica* is generally the most common of the three taxa in Europe, but the relative frequencies of the taxa vary between regions (Krebs et al., 2010). Few samples collected of *F. sachalinensis* in the present study may reflect a lower frequency of this species compared to the other two taxa in Norway. Also in other parts of Europe, *F. sachalinensis* is less frequent and seems to be less invasive, than *F*. *japonica* and *F*. × *bohemica* (Krebs et al., 2010; Mandák et al., 2005; Tiébré, Bizoux, et al., 2007). This may be due to the lower potential of *F. sachalinensis* to regenerate from rhizome fragments (Bímová, Mandák, & Pyšek, 2003).

The taxonomic identification based on morphology corresponded with the results of the molecular analyses (AFLP and SSR) as well as with the results of the flow cytometric analysis. AFLP analysis clearly clustered the samples corresponding to morphological identification. The diagnostic SSR marker, KW6, was detected in all samples identified as *F*. × *bohemica* or *F. sachalinensis*, but not in samples identified as *F*. *japonica*. The ploidy levels found for the taxa in the present study are the most common ploidy levels for these taxa in Europe (Krebs et al., 2010; Mandák et al., 2003). This indicates that morphological characters can be used to distinguish between the taxa in Norway. However, morphological variations within the taxa could confuse identification, especially when experience with the different taxa is lacking. Distinguishing *F*. *japonica* and *F*. × *bohemica* by morphological means can be even more challenging in regions where hybridization and backcrossing occur (Tiébré, Bizoux, et al., 2007).

This is the first genetic diversity study of the invasive *Fallopia* taxa in Norway. Extremely low levels of genetic variation were uncovered within the taxa in the present study. In terms of *F*. *japonica*, the present results corresponded with most previous studies in Europe (Hollingsworth & Bailey, 2000a; Krebs et al., 2010; Mandák et al., 2005; Tiébré, Bizoux, et al., 2007), in that no genetic variation was found within this species. Furthermore, the Norwegian *F*. *japonica* samples were the same AFLP genotype as the *F*. *japonica* reference sample from the UK, indicating that the octoploid male‐sterile clone that is dominant in Europe and North America (Gaskin et al., 2014; Hollingsworth & Bailey, 2000a) is also widespread in Norway. This was supported by the flow cytometry results, confirming that all *F*. *japonica* samples were octoploid, and by observations of only male‐sterile flowers. The results for *F*. *japonica* differ from the results of Bzdęga et al. (2012, 2016), who detected some genetic variation within *F*. *japonica* in Poland by use of AFLP.

For *F. sachalinensis*, the AFLP analysis using five primer pairs did not reveal any genetic variation. The SSR analysis resulted in two different genotypes, one collected in southeast Norway, and one collected in northern Norway. However, as the SSR analysis method was aimed at taxonomic identification rather than genotyping, it is difficult to base conclusions about genetic variation on the results. Only two SSR markers were used, and PCR results were visualized by regular agarose gel electrophoresis, which is not an accurate method for fragment separation. Previous studies in Europe have found varying levels of genetic variation within *F. sachalinensis*, for example, by use of inter simple sequence repeats and random amplified polymorphic DNA (RAPD) (Krebs et al., 2010; Pashley et al., 2007).

While other European studies have detected high levels of genetic variation within *F*. × *bohemica* by use of methods such as AFLP (Bzdęga et al., 2012, 2016), RAPD (Hollingsworth & Bailey, 2000b; Krebs et al., 2010; Tiébré, Bizoux, et al., 2007) and isoenzyme variation (Mandák et al., 2005), only one AFLP genotype was detected in the present study, indicating no genetic variation within *F*. × *bohemica* in Norway. However, as for *F. sachalinensis*, the SSR analysis indicated that one *F*. × *bohemica* sample was a different genotype than the rest of the *F*. × *bohemica* samples. Differences in the leaf and flower morphology between the one stand and the other *F*. × *bohemica* stands supported this result. Unique genotypes could result from rare, sexual reproduction in Norway or may have been introduced to Norway as seeds or vegetative propagules. It is not uncommon to find more genetic diversity when using SSR analysis compared to AFLP (Medini, Hamza, Rebai, & Baum, 2005; Powell et al., 1996). SSR regions are highly variable due to the high frequency of slipped strand mispairing mutation during DNA replication and repair, which is the main mechanism creating SSRs (Tautz, 1989).

The different results obtained for *F*. × *bohemica* in the present study compared to previous studies could in principle be due to the methods for recording genetic variation. Previous studies have used various methods, for example, RAPD (Hollingsworth & Bailey, 2000b; Krebs et al., 2010; Tiébré, Bizoux, et al., 2007), isoenzyme variation (Mandák et al., 2005), and AFLP (Bzdęga et al., 2016; Gaskin et al., 2014). Furthermore, previous studies that have used AFLP to examine these taxa used different, and different numbers of, primer combinations compared to the present study, which could explain the different results (Bzdęga et al., 2012, 2016; Gaskin et al., 2014). A more likely explanation for the low genetic variation within *F*. × *bohemica* in Norway compared to in other European countries is that the invasive *Fallopia* taxa do not, or to a much lesser extent, reproduce sexually in Norway. This may be due to low autumn temperatures and short growing season preventing the production of mature seeds in the taxa. If mature seeds are produced, the low genetic variation indicates limited germination and seedling survival. Furthermore, sexual reproduction of these taxa in Norway may be limited by pollen availability. In the present study, *F*. × *bohemica* was the only species observed with hermaphrodite flowers, and a single, hermaphrodite, hexaploid genotype seems to be dominant in Norway. Hexaploid *F*. × *bohemica* can contribute to sexual reproduction (Bailey et al., 2009), but it has previously been shown that hexaploid *F*. × *bohemica* has irregular meiosis, resulting in aneuploid gametes and offspring and reduced pollen fertility (Bailey, 1994). Moreover, low genetic diversity may limit sexual reproduction of these taxa due to general self‐incompatibility (Bailey, 1994).

Climate warming may affect the reproductive strategies of invasive *Fallopia* taxa. Climate scenario projections for Norway indicate that temperature will increase for all seasons and that the length of the growing season will increase with 1–3 months during this century (Official Norwegian Reports 2010). These changes can increase the opportunities for invasive *Fallopia* taxa to reproduce sexually in northern regions. Extension of the frost‐free period in the autumn may have increased germination of *F*. × *bohemica* in the Quebec region in Canada (Groeneveld et al., 2014) and may also have contributed to increased seed production in *F*. *japonica* in the UK (Bailey et al., 2009). Hermaphrodite *F*. × *bohemica* is a potential pollen source for the male‐sterile parental species and could play an important role for whether new, invasive genotypes can be produced under future warmer climate in Norway. Targeted eradication of *F*. × *bohemica* can therefore be a means to reduce this potential future effect of climate warming.

## CONFLICT OF INTEREST

None declared.

## AUTHORS' CONTRIBUTIONS

All authors contributed to the conception of the ideas and/or the design of the study. AKH and BWO acquired the samples. AE and AKH analyzed the data. AE, AKH and MBB interpreted the data and led the writing of the manuscript. All authors contributed critically to the drafts and gave final approval for publication.

## DATA ACCESSIBILITY

Data is archived at the data repository of the Norwegian Institute of Bioeconomy Research (NIBIO). https://nibiord.bibsys.no/jspui/handle/123456789/46
